# The reciprocal relation between stigma and suicidality in a sample of patients with affective disorders

**DOI:** 10.1192/j.eurpsy.2021.470

**Published:** 2021-08-13

**Authors:** L. Giannelli, G. Cristilli, L. Marone, G. De Felice, A. Carello, M. Di Vincenzo, V. Sollo, A. Di Cerbo, V. Giallonardo, G. Sampogna, V. Del Vecchio, M. Luciano, A. Fiorillo

**Affiliations:** Department Of Psychiatry, University of Campania “Luigi Vanvitelli”, Naples, Italy

**Keywords:** Stigma, Suicide, sucideprevention

## Abstract

**Introduction:**

Suicide is one of the major public health concerns worldwide, currently listed as the 15th most common cause of death. Mental illness stigma may contribute to suicidality and is associated with social isolation and low self-esteem among people with affective disorders.

**Objectives:**

The aim of the present study is to assess, in a sample of people with affective disorders, whether high levels of internalized stigma are associated to suicidal thoughts and behaviours.

**Methods:**

60 outpatients diagnosed with depression or bipolar disorder according to DSM-5 have been recruited. Suicidal behaviours and ideation were assessed through the Columbia Suicide Severity Rating Scale (C-SSRS); internalized stigma through the Internalized Stigma of Mental Illness (ISMI) scale. Socio-demographic characteristics have been collected through an ad hoc schedule.

**Results:**

62.9% of the sample was female, with a mean age of 45.7 (±14) years. About half of the sample had a diagnosis of major depression (54.8%). Patients with suicidal ideation reported higher score at ISMI “alienation” subscale (p<0,05), compared to those without suicidal ideation. Patients with a previous history suicide attempts reported higher score at “alienation” and “social withdrawal” ISMI subscales (p<0,05). Moreover, “alienation” ISMI subscale significantly correlated with suicidal ideation and behaviours (p<0,01).

**Conclusions:**

These results are in line with the available literature, highlighting that stigma and suicidality are strongly correlated. This underline the importance of interventions at addressing internalizing stigma, in particular to those with previous suicidal attempts and with an active suicidal ideation.

**Disclosure:**

No significant relationships.

